# P-258. Evaluation of Statin Prescriptions for People with HIV Over the Age of 40 in a Southeastern Non-Urban Ryan White HIV/AIDS Clinic

**DOI:** 10.1093/ofid/ofaf695.479

**Published:** 2026-01-11

**Authors:** Parmida Parsa, Erin Rogers, Feng Liu, Kathleen A McManus

**Affiliations:** University of Virginia, Charlottesville, VA; University of Virginia, Charlottesville, VA; University of Virginia, Charlottesville, VA; University of Virginia, Charlottesville, VA

## Abstract

**Background:**

Cardiovascular disease is a leading cause of morbidity and mortality in patients with HIV (PWH). Statin therapy reduces this risk, particularly in individuals over 40 years old, as demonstrated by the recent REPRIEVE trial. We examined statin prescription rates at a Ryan White HIV/AIDS Program (RWHAP) clinic in 2023 and 2024 and identified patient characteristics associated with a statin prescription in 2024.

**Figure:**
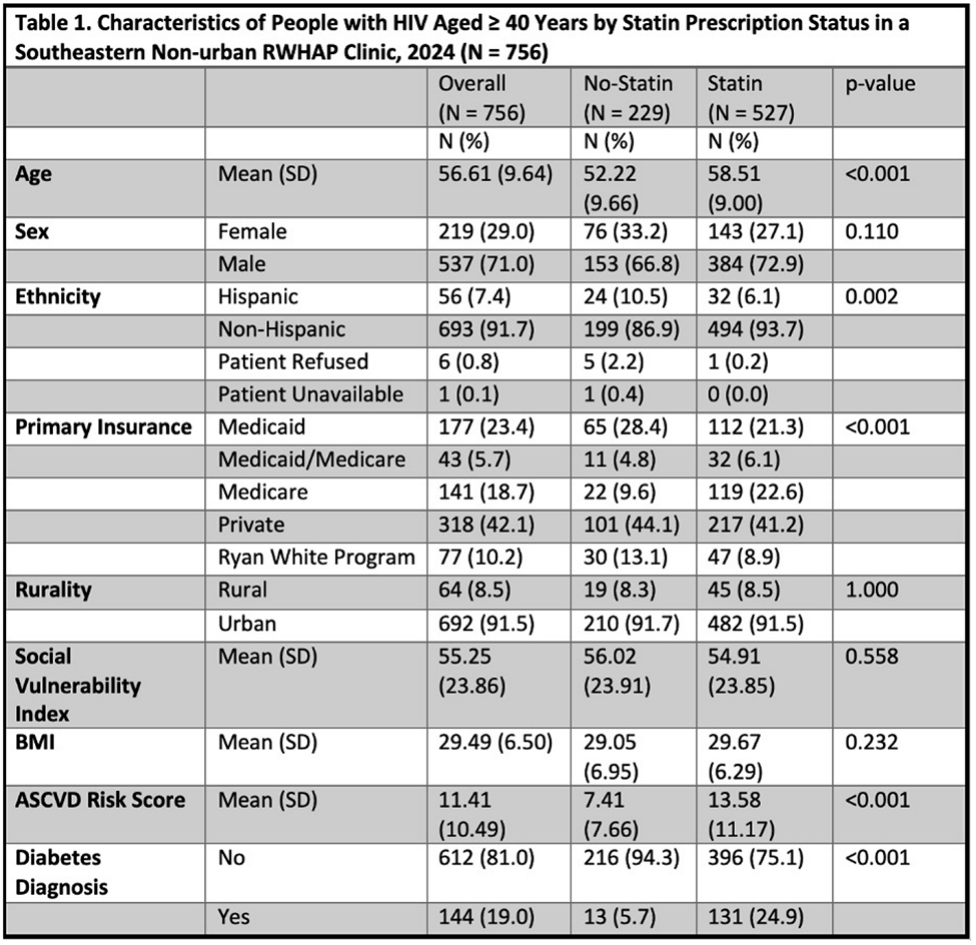

**Methods:**

The eligible population was PWH aged ≥ 40 who received care at a southeastern non-urban RWHAP Clinic in 2023 (N=764) and 2024 (N=756). From electronic health records, we collected demographic information (age, sex, ethnicity, insurance coverage, rurality), clinical information, and medications prescribed. We calculated the percent change in statin prescribing from 2023 to 2024 and identified characteristics associated with a 2024 statin prescription using descriptive statistics, t-tests for continuous variables, and chi-square tests for categorical variables

**Results:**

Statin prescription increased from 50% (383/764) in 2023 to 70% (527/756) in 2024, representing a 40% relative increase. PWH with statin prescriptions were older at 58.5 as compared to 52.2 for those without statin prescriptions (p < 0.001; Table 1). Hispanic PWH were less likely to be prescribed a statin than non-Hispanic PWH (p = 0.002). PWH with Medicare were more likely to be prescribed a statin while PWH with Medicaid or who were uninsured with Ryan White Program were less likely (p < 0.001). PWH with diabetes (p < 0.001) and higher ASCVD risk scores (p < 0.001) were more likely to be prescribed a statin. Prescription of a statin did not vary by sex, rurality, BMI, or social vulnerability index.

**Conclusion:**

The 40% relative increase in statin prescription between 2023 and 2024 demonstrates a high uptake of recent national guidelines about statins for primary cardiovascular prevention in PWH. Certain subpopulations (Hispanic PWH, PWH with Medicaid, and uninsured PWH with Ryan White Program coverage) had lower rates of statin prescription. These groups could have lower uptake due to lack of provider discussion, declining the prescription, or other reasons. More research is needed to optimize cardiovascular health for PWH.

**Disclosures:**

All Authors: No reported disclosures

